# Using Hypnotic Suggestion to Model Loss of Control and Awareness of Movements: An Exploratory fMRI Study

**DOI:** 10.1371/journal.pone.0078324

**Published:** 2013-10-21

**Authors:** Quinton Deeley, Eamonn Walsh, David A. Oakley, Vaughan Bell, Cristina Koppel, Mitul A. Mehta, Peter W. Halligan

**Affiliations:** 1 Cultural and Social Neuroscience Research Group, Forensic and Neurodevelopmental Sciences, Kings College London, Institute of Psychiatry, London, United Kingdom; 2 Division of Psychology and Language Sciences, University College London, United Kingdom; 3 Cultural and Social Neuroscience Research Group, Centre for Neuroimaging Sciences, Kings College London, Institute of Psychiatry, London, United Kingdom; 4 Department of Medicine, Imperial College London, South Kensington Campus, London, United Kingdom; 5 School of Psychology, Cardiff University, United Kingdom; University Medical Center Groningen UMCG, Netherlands

## Abstract

The feeling of voluntary control and awareness of movement is fundamental to our notions of selfhood and responsibility for actions, yet can be lost in neuropsychiatric syndromes (e.g. delusions of control, non-epileptic seizures) and culturally influenced dissociative states (e.g. attributions of spirit possession). The brain processes involved remain poorly understood. We used suggestion and functional magnetic resonance imaging (fMRI) to investigate loss of control and awareness of right hand movements in 15 highly hypnotically suggestible subjects. Loss of perceived control of movements was associated with reduced connectivity between supplementary motor area (SMA) and motor regions. Reduced awareness of involuntary movements was associated with less activation in parietal cortices (BA 7, BA 40) and insula. Collectively these results suggest that the sense of voluntary control of movement may critically depend on the functional coupling of SMA with motor systems, and provide a potential neural basis for the narrowing of awareness reported in pathological and culturally influenced dissociative phenomena.

## Introduction

The normal sense of being able to initiate and control movements is altered or lost in a variety of neuropsychiatric disorders [1]. For example, medial frontal and corpus callosum lesions are associated with the condition of ‘anarchic’ hand, whereby the limb produces stereotypic movements independent of the patient’s intentions [1]. Schizophrenia, focal epilepsy and right parietal lobe lesions are associated with the related but distinct condition of ‘alien control’, in which the patient reports that their actions are not generated by themselves but by a force or entity outside the self [1,2]. ‘Anarchic hand’ and ‘delusions of alien control’ involve loss of perceived control of movement, but the patient is typically both aware of the limb movement and that it is their limb that is moving without their voluntary control. As such, the experience of agency or perceived control of limb movement is lost, but ownership of the limb and awareness of limb movement are retained.

Involuntary movements (i.e. movements not attributed to the exercise of the agent’s will) can, however, also be accompanied by apparent narrowing or loss of awareness, as exemplified by psychogenic non-epileptic seizures defined as “episodes of altered movement, sensation, or experiences resembling epileptic seizures… not associated with ictal epileptiform discharges but which instead have a psychological origin” [3]. Awareness is partially or fully lost in up to 50% of patients [3]. As dissociative disorders these involve a “disruption of the usually integrated functions of consciousness, memory, identity or perception of the environment” [4] understood as “an involuntary response to emotional, physical, or social distress” [3]. Involuntary movements associated with narrowing or loss of awareness also occur in culturally-influenced dissociative phenomena linked to spirit possession, mediumship, and shamanism, which have been widely reported across different cultures and periods of history [5-9]. Nevertheless, whilst involuntary movements with or without loss of awareness are found in a variety of neuropsychiatric syndromes and culturally influenced alterations in experience and behaviour, the cognitive and neural processes underpinning these phenomena remain largely unknown [1,3]. 

One experimental approach to investigating voluntary control of movement and its loss in the laboratory involves employing hypnotic suggestion in healthy, non-clinical participants [10]. This approach was first used in combination with neuroimaging to investigate the neural correlates of experiences of alien control in a PET study that involved suggested misattributions of self-generated movements [11]. Six highly hypnotically suggestible male participants (scoring 9 to 12 on The Harvard Group Scale of Hypnotic Susceptibility: HGSHS) were hypnotised throughout a PET scanning session whilst experiencing repetitive vertical movements of the left arm produced (i) voluntarily (Active Movement condition), or (ii) by a suggestion that consisted of telling the participant that a pulley would be used to move the arm up and down when in fact the pulley was not activated (Deluded Passive Movement condition). The Deluded Passive movements were associated with significantly greater activations in bilateral cerebellum and parietal cortex relative to Active Movement. 

In that study it was proposed that the administration of a suggestion for external control prevented or disrupted the feedforward inhibition of sensory processing of self-generated actions that would otherwise occur according to the standard ‘forward model’ of motor control [12-14]. Failure to attenuate sensory processing for what in reality is self-generated movement was proposed to underlie the observed increases in cerebellar-parietal activity in the Deluded Passive condition. However, increased cerebellar-parietal activity during ‘Deluded Passive’ movements could also represent the neural correlates of the heightened sensory salience of unexpected movements, rather than the experience of involuntariness *per se*. Indeed, these activations were also seen during a Passive Movement condition, when movement was produced passively via the operation of a pulley system [11]. Hence, an alternative hypothesis is that the experience or feeling of the involuntariness of suggested ‘alien control’ results from changes occurring at the level of executive motor planning and intention. 

In healthy individuals, the supplementary motor area (SMA) is active during motor planning and the intention to move [15-18]. The SMA is active both before the onset and during movements subjectively reported to be self-initiated or controlled [19]. SMA activity precedes conscious awareness of the decision to initiate a given action [20], while stimulation of the SMA via electrodes is associated with an urge to move [21]. Normal motor output is also accompanied by connectivity between the SMA and systems involved in the implementation of the actions (M1, basal ganglia, and cerebellum) [19,22]. Consequently, reduced activation of SMA or reduced functional connectivity with other regions involved in action implementation (such as premotor cortex, M1, S1, and cerebellum) are likely candidates for mediating the loss of perceived voluntary control following attributions of self-generated actions to a source other than the self – as occurs in association with pathological and culturally influenced dissociative phenomena, delusions of alien control, or suggestions of involuntary movement. 

Also, as far as we are aware no previous studies have combined suggestion and neuroimaging to explicitly examine the neural correlates of suggested involuntary movement accompanied by loss of awareness (to model symptoms and experiences reported by patients experiencing psychogenic non-epileptic seizures [3] or culturally influenced dissociative states [8,9]). 

We predicted that reduction in subjective awareness of involuntary movements would be associated with reduced activation of superior parietal cortex (BA 7), a brain region involved in the integration of somatosensory and visual information during visuomotor coordination of hand movements, in addition to a more general awareness of the bodily self [23,24]. We also expected that reduced bodily awareness would be associated with reduced activation of the insula, a key brain region supporting somatosensory awareness [25,26].

To investigate the functional anatomy of *loss of limb control* and *awareness of movements* in highly hypnotically suggestible subjects, we employed a controlled design using functional magnetic resonance imaging (fMRI). Suggestions were used to create selective changes in behaviour that were experienced as involuntary and which reproduced symptoms or experiential changes analogous to those observed in psychopathology or culturally influenced dissociative states [6,10]. For the purposes of these experiments participants were required to move a joystick with their right hand under a variety of suggested conditions following a standardised hypnotic induction procedure. These included:

a. normal voluntary movement;
b. involuntary movement with preserved awareness (such as occurs in passivity phenomena in schizophrenia and in association with psychogenic non-epileptic seizures and other dissociative involuntary movements); and
c. involuntary movement with reduced awareness (modelling psychogenic non-epileptic seizures and other dissociative involuntary movements accompanied by loss of awareness).

To summarise, the main hypotheses were that:

1. involuntary movement relative to voluntary movement would be associated with decreased activity of the SMA and reduced functional connectivity of the SMA with motor regions involved in movement implementation [27];
2. reduced awareness relative to preserved awareness of involuntary movements would be associated with reduced activation of superior parietal cortex (BA 7) and insula;
3. involuntary relative to voluntary movement would be associated with significantly reduced perceived control but not awareness of hand movements; and
4. the introduction of a suggestion of reduced awareness would be additionally associated with significant reductions in subjective measures of awareness.

Also, whilst we did not employ targeted suggestions to directly modify the sense of hand ownership, we acquired self-ratings of ownership to test whether suggested loss of control also affects the sense of hand ownership [28]. 

## Methods

### Experimental Conditions and Contrasts

The study involved seven experimental conditions. The first four conditions focused on voluntariness and awareness and are presented in the current paper. 

The 3 key contrasts (see Table 1) are:

I. Main effect of hypnosis: nhyp-vol vs. hyp-vol
II. Main effect of voluntariness: hyp-involA vs. hyp-vol
III. Awareness contrast: hyp-involA vs hyp-involNA

Three subsequent conditions varied attributions about personal and impersonal causes of involuntary movement. These findings are presented in a separate paper. 

**Table 1:** The four experimental conditions, the abbreviated name and the focus of the suggestion for each condition.

**Condition Name**	**Abbreviation**	**Suggestion**
**1**. Non-Hypnosis (normal alert state)	nhyp-vol	Before hypnosis - normal movement
**2**. Hypnosis-Involuntary	hyp-involA	During hypnosis – “hand moves all by itself”. Normal awareness
**3**. Hypnosis-Involuntary-No-Awareness	hyp-involNA	During hypnosis – “hand moves all by itself”. Reduced awareness of movement, body and surroundings
**4**. Hypnosis-Voluntary	hyp-vol	During hypnosis - normal movement

The three contrasts of interest are the ‘hypnosis contrast’ ((Nr. 1 vs. 4)), the ‘voluntariness contrast’ ((Nr. 2 vs. 4)) and the ‘awareness contrast’ ((Nr. 2 vs. 3)).

Experimental conditions were presented in a randomized order across subjects, with the exception of a) the nhyp-vol condition, which always occurred prior to induction of hypnosis; and b) the hyp-vol condition, which was the first condition presented following hypnotic induction. In order to control for effects of hypnosis on baseline measures of control, ownership, and awareness of movements, and potential effects of hypnosis *per se* on brain function, the hyp-vol condition was used in subsequent contrasts to determine the effects of suggested involuntariness on brain activity. In addition, the hyp-vol condition was a suitable contrast condition because the degree of movement did not significantly differ between hypnosis conditions (see below). 

### Participants

The research was approved by the Ethics Committee of the Institute of Psychiatry, Kings College London (040/02). Participants provided their written informed consent to participate in this study. Thirty-three highly hypnotically suggestible individuals (14 Male/ 19 Female) were recruited as possible participants from databases of volunteers previously tested on The Harvard Group Scale of Hypnotic Susceptibility (Form A) (HGSHS:A) [29] at either University College London (UCL, N = 16) or the Institute of Psychiatry (IoP, N = 17). Their HGSHS scores ranged from 8 to 12 with a mean of 10.06 (SD 1.06). All 33 were screened using the same protocol as that used in the neuroimaging study (described below) except that participants were required to make hand movements with their right hand similar to those involved in moving a joystick from side to side whereas an actual joystick, held by the participant in their right hand, was present in the scanner.

This screening was carried out in a small, normally lit experimental room at either UCL (N= 31) or at the IoP (N = 2) with participants sitting in a comfortable chair. Fifteen of these 33 potential participants went on to the scanning study. Reasons for exclusion of the remaining 18 were: did not experience reliable change in sense of ‘agency’ in response to suggestion (N = 7); not suitable for scanning [metal in body, grommets, too big for scanner, recent subdural haemorrhage] (4); not available (3); no/small overt motor responses (2); left handed (2). 

Of the 15 participants scanned 5 were male and 10 female, their mean HGSHS score was 9.8 (SD 1.08: range 8-12), their mean age was 33.67 (SD 11.91: Range 20-61 years). The majority were undergraduate or postgraduate students (8), while the remainder were members of a variety of professions. Eleven participants (7 female) were included in the final data analysis (three were excluded due to excessive drop-out of frontal MRI signal and one due to scanner data acquisition technical error). Their mean HGSHS score was 9.91 (SD=1.04, Range 9-12) and their mean age was 29.27 years (SD= 8.40, Range 20-47).

### Hypnotic induction and reversal

The hypnosis induction procedure was carried out with the participant lying in the scanner with their eyes open and fixating on a target consisting of a white crosshair projected on a black background. An experimenter administered suggestions (reported verbatim below, where ‘…’ indicates a pause). Continued fixation on the target was accompanied by suggestions of involuntary eye closure (“your eyes will begin to close all by themselves”) and the participant was asked to say “yes” when this had happened. The eye closure suggestions were combined with suggestions of muscle relaxation until the participant said “yes” after which the relaxation suggestions continued alone. The muscle relaxation sequence commenced with the face, progressing systematically throughout the body to the legs and feet. Finally there was a counting procedure (1-20) preceded by the suggestion that this would be accompanied by (i) a further deepening of the experience of relaxation, (ii) a reduced awareness of background sounds, and (iii) the ability to carry out all the things they would be required to do without it disturbing the state they had achieved at the end of the count. Participants’ eyes remained closed throughout their experience of hypnosis.

Reversal of hypnosis was achieved by a reversed counting procedure (20,) preceded by the suggestion that this would be accompanied at some point by the participant’s eyes opening and that they would be “wide awake … fully alert” at the end of the count. The participant was asked to say ‘yes’ when their eyes opened. The scripts used for the hypnotic induction and reversal stages are included verbatim in Oakley et al. [30] except that the ‘Special Place’ procedure was omitted in the present study. 

### Voluntary movement instructions and ‘Agency’ suggestions

In the briefing before the experimental procedure commenced, and in a practice run in the scanner, participants were reminded that *“though your arm may feel relaxed you will retain your grip and your hand will remain in contact with the joystick at all times”*, and *“in all movement tests your hand will not move during the instruction to ‘Rest’ – but will make the required movement when the instruction to ‘Move’ is given – though your experience of that movement will vary.”* They were reminded to continue to hold the joystick in their right hand prior to each scan.

i) For the ‘voluntary’ movement condition before [nhyp-vol] and during hypnosis [hyp-vol] the instructions to the participant were: *“In a moment you will hear recorded instructions at regular intervals. The instruction will say, “REST”, which simply means not attempting to use or prepare to move your right hand; or “MOVE” which means move the joystick to the **right** and then to the **left** once with your right hand each time. The instructions will come at regular intervals. Don’t guess what is coming, just listen and follow the instructions to the best of your ability”*. Both nhyp-vol and hyp-vol were carried out with the participant’s eyes closed.
ii) For the hypnotic involuntary movement condition [hyp-involA] the instructions combined with a suggestion were *“Just remain as relaxed and hypnotised as you are now… As before you will hear recorded instructions at regular intervals. When you hear the word “REST” do nothing- just relax. When you hear the word “MOVE” your right hand will move **all by itself** … and will move the joystick to the **right** and then to the **left** once each time. Your right hand will make this movement all by itself; you will feel no control over when your right hand is going to move but you will be clearly aware of the movement of your hand and of the joystick when it occurs. You will remain calm and relaxed during these movements of your hand. The instructions will come at regular intervals. Don’t guess what is coming, but at all stages listen to the recorded instructions.”* At the end of this condition the suggestion was removed as follows *“Your right hand no longer moves of its own accord – it is back under your control – your right hand is back to normal again. Say “yes” when this has happened.”*
iii) For the involuntary movement with loss of awareness condition [hyp-involNA] the instructions combined with suggestions were: *“Just remain as relaxed and hypnotised as you are now… As you do so you begin to lose awareness of your own body and your surroundings – it is as though your body ceases to exist for you and you are unaware of the positions of your arms and your legs – unaware of your hands and your fingers – unaware of your own actions and any movements you might make. You remain aware of being yourself, but have no experience of your body or the world around you. You will however be aware of the spoken instructions. You will remain calm and relaxed throughout. As before the recorded instructions will occur at regular intervals. When the word “REST” is spoken your body will not respond – you will simply remain relaxed. When the word “MOVE” is spoken your right hand will move **all by itself** … and will move the joystick to the **right** and then to the **left** once each time but you will **not be aware** that it has done so. Your right hand will make this movement all by itself but you will not know that this has happened. You will have no control over when your right hand is going to move and no awareness of the movement of your hand and of the joystick when it occurs. The instructions will come at regular intervals. At all stages listen to the recorded instructions.”*

At the end of this condition the suggestions were removed as follows: *“Just focus your attention back to your body and as you do so you begin to regain your awareness of your own body and your surroundings – you become aware of the positions of your arms and your legs - aware of your hands and your fingers – aware of your own actions and any movements you might make. Your mind remains clear and in touch with your own internal thoughts and ideas and is reconnected with your body and your surroundings – and you are once more aware of your body.”* The participant was asked to say “yes” when normal awareness of body and surroundings had returned.

There were four additional conditions (making a total of eight conditions), which are not reported here. These included a repeat of the voluntary movement condition at the end of the scanning session and three additional agentive control conditions. 

### Motor Tasks and Induction of Alterations in Agency

Presentation of motor epochs and concurrent acquisition of fMRI data followed a block periodic design involving repeated alternation between non-movement (‘Rest’) and activation (‘Move’) epochs (30 second intervals) with 10 ‘Move’ or ‘Rest’ instructions per epoch administered at 3 second intervals. Each block comprised 10 epochs (five ‘Move’, five ‘Rest’) so that each block was of 5 minutes duration. The motor task was therefore ‘invariantly instructed’ rather than ‘free choice’, given that the movements occurred in response to a standardised instruction (‘Move’) [31].

During scanning, joystick displacement was carefully monitored. A condition was repeated if no joystick movement was observed, resulting in the repetition of three conditions out of 88 (across all participants and conditions). The position of the joystick was recorded throughout each condition and the standard deviation of the position was used to index joystick displacement amplitude for the Move trials [32]. 

At the end of each experimental block, participants verbally rated their subjective experience of the movement of their right hand with respect to: (i) awareness (from ‘0’ to ‘10’, where ‘0’ means ‘you had no awareness of your hand and its movement’ and ‘10’ means ‘you had full normal awareness of the movements of your hand’); control (‘0’ means ‘you had no part in initiating or controlling the movement of your hand’ and ‘10’ means ‘you and you alone initiated and controlled the movement in response to the instructions’); and (iii) ownership (‘0’ means ‘you do not experience the moving hand as being your hand – it does not feel like your hand’ – and ‘10’ means ‘you have the normal sense of the moving hand being your own hand’). Two additional ratings were taken of (i) the strength of emotion reaction participants felt during each experimental block (‘0’ means ‘you felt no emotional reaction associated with the instructions or suggestions given about the hand movement’ while ‘10’ means that ‘the instructions or suggestions given about the hand movement created a very strong emotional reaction’) and (ii) subjective ‘depth of hypnosis,’ also rated from 0-10 [30].

### Image Acquisition Parameters

Imaging data were acquired at 3 Tesla (3T) using a GE Signa HDx MRI scanner at the Centre for Neuroimaging Sciences, Kings College London UK. Functional MRI examinations were conducted using gradient echo, echoplanar imaging (EPI) with the following scanner parameters: repetition time = 2000 msec; echo time = 30 msec; RF flip angle = 80 degrees; Slice orientation = near-axial, aligned to the anterior-posterior commissure; number of slices = 40, interleaved acquisition; slice thickness = 3 mm; slice gap = 0.3 mm; acquisition matrix size = 64 x 64. For each of the nine experimental blocks (conditions), a total of 150 functional images were acquired continuously.

### Neuroimaging data analysis

Functional images were processed and analysed in SPM5 (Wellcome Trust Centre for Neuroimaging, London, UK; http://www.fil.ion.ucl.ac.uk/spm). All images were initially realigned to first image and then their mean image. The mean image was spatially normalized to the SPM5 EPI template and spatially smoothed (8 mm FWHM Gaussian kernel), and high-pass filtered (128 s). The general linear model (GLM) was then used to generate parameter estimates of activity at each voxel, for each of the experimental conditions using single-subject models. These included a condition coding the onsets and durations of the movement blocks (convolved with the canonical haemodynamic response function) and the six motion parameters derived from the realignment procedure. A flexible-factorial ANOVA group analysis (unequal variances) was conducted on contrast images for movement versus rest from the single-subject analyses, with condition as a fixed effect and subject as a random effect. Group brain activation maps were calculated for ‘movement’ versus ‘rest’ to confirm significant engagement of the motor network for each condition and contrasts between conditions were generated to test the effects of hypnosis and targeted suggestions. Statistical significance was defined at the cluster level (p<0.05 after multiple comparisons correction, with a voxelwise threshold of p<0.001).

### Functional connectivity analysis

To establish the nature of interactions between different brain areas that mediate joystick movements under the different experimental conditions, we also performed a psychophysiological interaction analysis [33]. The first step was the selection of the seed region for SMA [18]. While some individual subjects activated the SMA, the movement versus rest contrast in the non-hypnosis condition did not produce a significant activation at the group level. The coordinates for the SMA seed (-2, -12, 53) were therefore calculated from the mean values from two studies with similar motor tasks to our study, which were -2, -14, 52 [34] and -2, -10, 54 [22]. The seed for the PPI analysis for each participant was defined as the eigenvalue for a 6mm radius sphere around the peak activation nearest the SMA seed coordinates. In order to ensure the data were from the same functional locale across all individuals, we excluded participants for whom the Euclidian distance between the peak activation voxel per condition closest to the seed coordinates for SMA exceeded 6 mm. Therefore, the PPI analysis for SMA included seven participants (four excluded).

For the PPI analyses the single-subject models included regressors for the seed region, the task design (‘movement’ versus ‘rest’), their interaction as described in Friston et al., (1997) [35] and the six movement parameters. The PPI contrast image was entered into a flexible factorial ANOVA model including all eight experimental conditions. We then tested changes in connectivity in the contrast of hyp-vol and hyp-involA across the whole brain. Given that our sample size was smaller for the PPI analyses because we excluded participants whose seed voxels lay outside our defined functional locale (see above), we used a voxel-wise threshold of p < 0.01, with statistical inference based on a cluster statistics threshold of p<0.05 corrected for multiple comparisons. 

## Results

### The effects of hypnosis and specific suggestions on joystick displacement

The mean joystick movement values (standard deviation in parentheses) for the four experimental conditions were nhyp-vol 21.8 (SD = 6.4); hyp-vol 18.8 (SD = 6.9); hyp-involA 16.6 (SD = 9.4); and hyp-volNA 12.3 (SD = 8.9). There was a main effect of hypnosis: movement amplitude in the nhyp-vol (non-hypnosis) condition (mean=21.8; SD=6.4) was significantly greater than the movement amplitude in the hyp-vol (hypnosis) condition (mean=18.8; SD=6.9); t(9)=2.55; p=0.031. Furthermore, participants moved the joystick to the same extent regardless of whether the suggestion was to move the right hand voluntarily (hyp-vol) or that the hand “moved all by itself” (hyp-involA) mean=16.6; SD=9.4); t(9)=1.18 p=0.268). Similarly the difference in joystick displacement did not reach statistical significance for the hyp-involA and the hyp-involNA (mean=12.3; SD=8.9); t(9)=1.966; p=0.081, although it could be argued that there was a trend towards less movement in the hyp-involNA. The joystick displacement data for one participant were unavailable for technical reasons.

In order to test if subtle differences in movement affected the brain imaging results presented below, the participants with the largest differences between the two conditions were identified and the imaging results tested without them. In addition, correlations between the imaging findings and joystick movement were performed (see below). 

### Self-ratings

Depth of hypnosis: For the three hypnosis conditions hyp-vol, hyp-involA and hyp-involNA, the mean self-rated depths of hypnosis were 7.8 (SD=1.5), 7.7 (1.5) and 7.6 (1.2) respectively, and were as expected, significantly higher than the nhyp-vol condition (mean=1.7; SD=2.1); F(3,30)=60.6; p<0.0001. For the same three hypnosis conditions, direct contrasts between them showed that there were no significant differences in depth of hypnosis (all p>0.640). 

Self-ratings associated with the awareness, control and ownership of the right-hand and its movements following the suggestions are given in Table 2. For each of these ratings there was a main effect of hypnosis (nhyp-vol vs hyp-vol); when in the non-hypnosis condition as opposed to the hypnosis condition, participants reported significantly higher ratings for awareness t(10)= 3.75; p=0.004; control t(10)= 2.76; p=0.020 and ownership t(10)= 2.73; p=0.021. 

**Table 2:** Subjective ratings (0 to 10 scale; 0=none; 10=full) for awareness, control, ownership and emotion for the 4 experimental conditions (group means; standard deviations are given in brackets).

	**hyp-vol**	**hyp-involA**	**hyp-involNA**	**nhyp-vol**
**Awareness**	8.1 (1.2)	7.6 (1.8)	5.1 (2.4)	9.5 (0.9)
**Control**	9.2 (0.8)	5.7 (2.1)	4.0 (2.7)	9.6 (0.7)
**Ownership**	9.0 (1.2)	6.0 (2.3)	3.7 (2.9)	9.8 (0.4)
**Emotion**	1.8 (2.4)	2.5 (2.2)	2.7 (2.0)	2.6 (2.7)

The suggestion that a participant’s hand would move “all by itself” (hyp-involA) as compared to it moving voluntarily (hyp-vol) was associated with significantly reduced ratings of control t(10)= 5.17; p<0.001 and ownership t(10)= 4.04; p=0.002. There was no difference in awareness ratings for the hyp-involA condition t(10)= 0.971; p=0.355.

When it was suggested, “you will not be aware of your hand or its movements…” (hyp-involNA vs hyp-involA), participants reported significant reductions in feelings of awareness (t(10)= 3.65; p=0.004), control (t(10)= 3.19; p=0.010) and ownership (t(10)= 3.28; p<0.001). In other words, the reduced awareness suggestion produced a profound effect on people’s subjective experience of their movements, body, and surroundings.

Planned t-tests (two tailed) were performed for the three contrasts of interest for emotion ratings, and revealed that there were no significant differences between conditions:

I. Main effect of hypnosis: (nhyp-vol vs hyp-vol); t(10)=1.057 ; p=0.321.
II. Main effect of voluntariness: (hyp-involA vs hyp-vol); t(10)=1.550 ; p=0.152.
III. Awareness contrast: (hyp-involA vs hyp-involNA); t(10)=0.602 ; p=0.561.

### fMRI Data: General Linear Model Analyses

#### (i): Task-related engagement of the motor system in the non-hypnosis condition

**Figure 1:**
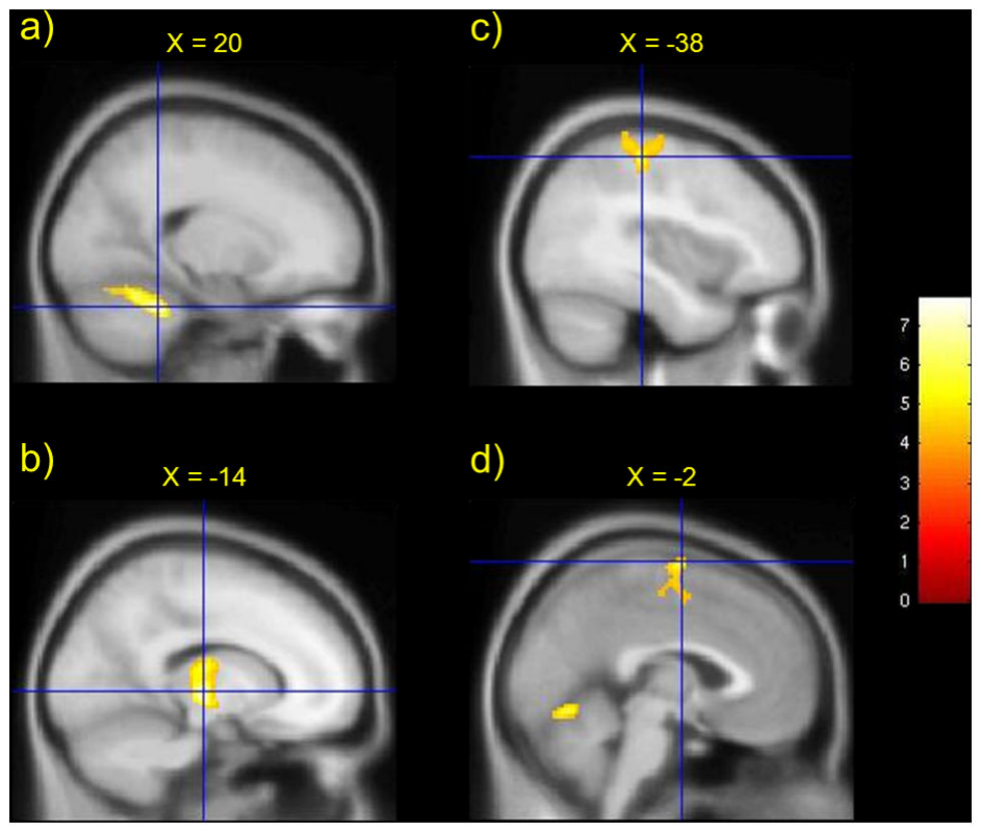
Performance of the motor task (joystick movement) in the non-hypnosis condition (nhyp-vol condition) engaged established components of the ‘motor network’, including a) right cerebellum b) left thalamus c) left postcentral gyrus (M1) and d) left SMA. Crosshairs show peak activation at each cluster.

First we identified activations produced by movement of the joystick in the non-hypnosis condition. These analyses showed that classic motor regions and networks (including left M1, left SMA and right cerebellum) were significantly activated by right hand movements (Figure 1). Significant activations were also observed in left thalamus and left postcentral gyrus. 

#### (ii): Main effect of hypnosis during joystick movement

Next we tested for any changes in brain activity during joystick movement in the hypnotic state (hyp-vol; i.e. without suggestions of involuntary movement) as compared with the non-hypnosis condition (nhyp-vol). This analysis revealed no significant differences in brain activity. 

#### (iii): Voluntary versus involuntary movement during hypnosis, with preserved awareness

Following the induction of hypnosis, there were no significant differences in brain activity during voluntary (hyp-vol) compared to involuntary (hyp-invol) movement. The similarity between these two conditions was supported by a correlational analysis [36] which showed that most voxels within the motor cortex and cerebellar activation clusters had reliability of Intraclass Coefficient > 0.5 (data not shown). 

#### (iv): Changes in brain activity associated with reduced awareness of movement

**Figure 2:**
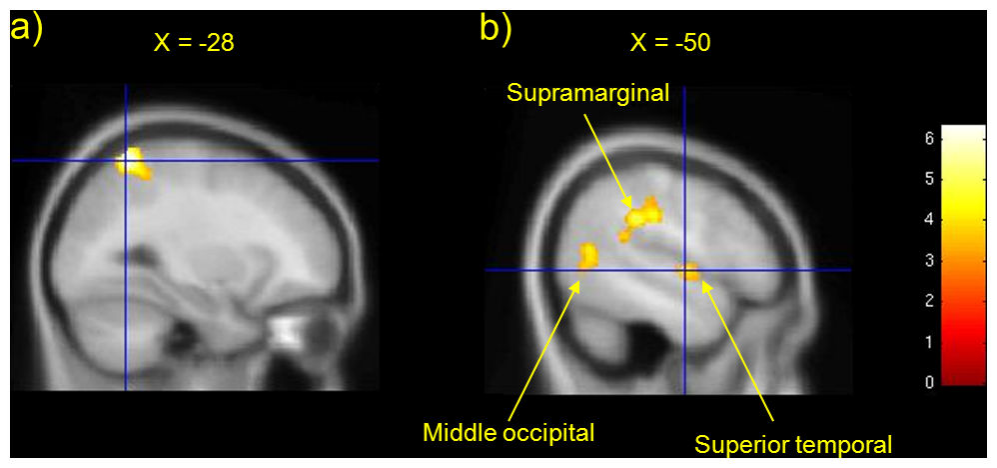
Suggested loss of awareness (hyp-involA versus hyp-involNA , 2>3)) was associated with reduced activation in a) left SPL (BA 7) and IPL (BA 40) and b) left supramarginal, left middle occipital and left superior temporal gyri (p<0.001).

The contrast of brain activity during involuntary movement with normal awareness and involuntary movement with reduced awareness of body, movement and surroundings (hyp-involA vs. hyp-invol-NA) showed that reduced awareness was associated with significantly decreased activity in left inferior and superior parietal lobules as well as left superior temporal and visual areas (Figure 2 and Table 3). The results remained significant after removal of the two participants who moved the joystick the least in the reduced awareness condition. Also, the results did not correlate with movement; specifically, Spearman’s correlation confirmed that there were no significant relations between the joystick movement parameters and insula signal change (Spearman’s rho = 0.30; p=0.41) or somatosensory cortex change (Spearman’s rho = 0.09; p=0.80). This suggests that subtle and non-significant reductions in joystick movements (and any changes in proprioceptive feedback associated with such reductions) could not explain the differences in brain activity between involuntary movement with and without awareness that we report. 

**Table 3:** Clusters of activations in a) during movement in the non-hypnosis condition compared to rest (nhyp-vol vs. Rest); b) Non-hypnosis condition vs. voluntary movement during hypnosis (nhyp-vol vs. hyp-vol) and c) involuntary movement with awareness vs. involuntary movement without awareness (hyp-involA vs hyp-involNA). Regions in bold type are the statistical peak of each cluster while regions shown in normal (non-bold) typeface refer to other significant voxels within the same cluster.

**Anatomical Region**	**Hemisphere**	**MNI coordinates**	**Cluster size**	**Z value**	**Cluster-levelp corrected**	**BA**
***a) nhyp-vol > Rest***						
**Cerebellum**	**Right**	**20, -44, -30**	**1402**	**7.71**	**0.000**	
**Thalamus**	**Left**	**-14, -18, 0**	**648**	**4.19**	**0.000**	**35**
Amygdala	Left	-20, -4, -10				34
Putamen	Left	-24, -10, 6				-
**Postcentral**	**Left**	**-38, -28, 56**	**669**	**3.74**	**0.000**	**3**
Precentral	Left	-30, -22, 54				4
**SMA**	**Left**	**-2, -6, 72**	**218**	**3.57**	**0.007**	**6**
Mid Cingulum	Left	-8, -6, 52				-
SMA	Right	2, -14, 60				6
**b) nhyp-vol vs hyp-vol**						
No significant differences						
**c) hyp-involA > hyp-involNA**						
**Superior Parietal**	**Left**	**-28, -58, 62**	**813**	**5.60**	**0.000**	**7**
Inferior Parietal	Left	-34, -40, 46				40
**Supramarginal gyrus**	**Left**	**-48, -36, 30**	**487**	**4.65**	**0.001**	**48**
Superior Temporal	Left	-50, -42, 20				41
**Mid Occipital**	**Left**	**-44, -66, 6**	**333**	**4.06**	**0.007**	**37**
Middle Temporal	Left	-48, -66, 12				37
**Superior Temporal**	**Left**	**-50, -8, 0**	**243**	**3.72**	**0.026**	**48**
Putamen	Left	-34, 0, 4				48
Insula	Left	-36, -2, -4				48

### fMRI data: functional connectivity (Psychophysiological Interaction (PPI) analysis)

**Figure 3:**
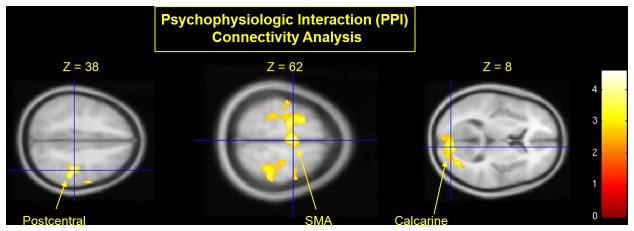
Functional connectivity analysis showed that following the induction of hypnosis, relative to the experience of voluntary movement, the suggested experience of involuntary movement(hyp-involA < hyp-vol) was associated with reduced connectivity between the SMA (seed = -2, -12, 53) and the right postcentral (M1) and calcarine visual cortex. Crosshairs show peak activations for each cluster; all thresholded at p<0.01.

We predicted that suggested involuntary movement would be associated with reduced functional connectivity between key regions involved in motor planning (SMA) and implementation (M1). Psychophysiological interaction [33] connectivity analysis showed reduced connectivity between SMA and other brain regions following suggested involuntary movement relative to voluntary movement (hyp-vol > hyp-involA) as detailed in Table 4. The SMA seed showed reduced connectivity with right M1 and right calcarine visual cortex (see Figure 3.b). 

**Table 4:** Psychophysiological interaction (PPI) connectivity analysis showing reduced connectivity between the SMA and other brain regions following suggested involuntary movement relative to voluntary movement (hyp-vol > hyp-involA): (SMA seed = -2, -12, 53) (p<0.01).

**Anatomical Region**	**Hemisphere**	**MNI coordinates**	**Cluster size**	**Z value**	**Cluster-levelp corrected**	**BA**
**Postcentral**	**Right**	**44, -26, 38**	**1728**	**3.81**	**0.000**	**3**
Precentral	Right	60, -6, 46		3.50		4
Supramarginal	Right	52, -34, 38		3.48		2
Inferior Parietal	Right	56, -38, 52		3.36		40
Superior Frontal		32, -8, 68		2.90		6
**SMA**	**Midline**	**0, -8 62**	**1721**	**3.66**	**0.000**	**6**
Paracentral Lobule	Left	-18, -12, 64				6
Mid-Frontal	Left	-24, 4, 60				6
Superior Frontal	Left	-24, 2, 68				6
**Calcarine**	**Right**	**10, -84, 8**	**1176**	**3.46**	**0.003**	**17**
Inferior Occipital	Right	34, -84, -4				19
Mid-Occipital	Right	34, -72, 10				37
Mid-Temporal	Right	38, -64, 10				37

Regions in bold type are the statistical peak of each cluster while regions shown in normal (non-bold) typeface refer to other significant voxels within the same cluster.

## Discussion

Functional magnetic resonance imaging (fMRI) was employed to investigate both the functional anatomy of loss of control and awareness of right hand movements in healthy, highly hypnotically suggestible subjects. The main findings were that:

- subjective ratings of control and awareness varied in accordance with the specific suggestions for loss of control and awareness when compared to voluntary movement following the induction of hypnosis;
- loss of voluntary control of hand movement was associated with reduced connectivity between SMA and components of the motor network involved in movement implementation; and
- reductions in movement-related activation were observed in the left superior parietal cortex and insula during involuntary movement with reduced awareness.

### Effects of hypnosis induction on subjective experience and movement related brain activity

Subjective experience as assessed by self-rating measures indicated that the hypnotic induction procedure produced a significant increase of depth of hypnosis for all conditions compared to the non-hypnosis condition with no significant differences in self-rated depth of hypnosis between hypnosis conditions. Compared to voluntary movement in the non-hypnosis condition (nhyp-vol), voluntary movement following the induction of hypnosis (hyp-vol) was associated with small but significant reductions in the perceived control, ownership and awareness of movements. However, there were no significant differences in brain activity during voluntary compared to involuntary movement. Compared to rest, voluntary movement in the non-hypnosis condition, and following the induction of hypnosis, was associated with increased activity in established brain regions involved in voluntary action, including bilateral SMA, contralateral premotor cortex, M1, S1 and thalamus, and ipsilateral cerebellum (Table 3a). Hence, the experimental paradigm elicited activity in brain systems known to be associated with voluntary movement. 

### Loss of voluntary control of movements

Compared to voluntary movements, targeted suggestions of involuntary hand movement (hyp-involA) were associated with significant reductions in the self-rated control and ownership but *not* awareness of hand movements as predicted. This reduction of the sense of hand ownership following suggestions of loss of control of hand movement supports the view that control and ownership of movements are closely linked phenomenologically and may be mediated by shared brain mechanisms [28]. Furthermore, relative to voluntary movement (hyp-vol) suggestions for loss of control and ownership of hand movement were accompanied by reduced connectivity between SMA and motor implementation regions in the absence of any differences in magnitude of BOLD signal. These results should be regarded as preliminary because of the small sample size employed for PPI (n=7). Nevertheless, the findings suggest that temporal correlation between the activity within brain regions involved in the initiation and execution of movements, rather than the magnitude of their activity, is responsible for the experience that movements are voluntary as opposed to involuntary. Future studies employing EEG could test whether perceived loss of voluntary control (i.e. involuntary movement) is associated with desynchronisation of midline frontal and motor regions. 

We also found that involuntary relative to voluntary movements were associated with reduced connectivity between SMA with visual cortical regions. The reason for these reductions in connectivity is unknown. However, while participants’ eyes were closed throughout the experiment, activation of visual cortical regions might be expected if visual imagery strategies were employed by participants while performing the task [37,38]. The relationship between visual imagery, perceived involuntariness of movements, and brain connectivity requires further investigation. 

Furthermore, we note that all changes in connectivity were ipsilateral with no evidence that contralateral connectivity was affected. This was in contrast to the GLM analysis, which showed the classic pattern of contralateral M1 and ipsilateral cerebellar activity during movement compared to rest (Figure 1). Loss of the sense of voluntary control may be related to changes in the contributions of each hemisphere to motor control. This could be tested in future studies – for example, by parametrically varying the level of perceived voluntary control of movement and correlating this with PPI to determine differential effects on hemispheric connectivity. 

### Loss of awareness of movement

Suggested loss of awareness relative to preserved awareness of involuntary movements was associated with significantly reduced self-ratings of awareness, control, and ownership of movements. At a neural level, loss of awareness was associated with significant reductions in activity in left parietal superior (BA 7) and inferior (BA 40) parietal cortices and supramarginal gyrus; temporal regions (superior, middle, and BA 37); and putamen and insula. These findings support our prior hypothesis that narrowing of awareness of body, environment and hand movements would be associated with reduced activity in parietal regions (BA 7) involved in representing the sense of body in space and in relation to limb movements, and reductions in insula activity associated with reduced somatosensory awareness. Reduced activity in visual processing regions (BA 37) may be associated with a reduction of vivid visual imagery consequent on suggestions of loss of awareness. 

While suggested involuntary relative to voluntary movement was associated with reductions in functional connectivity of SMA with motor implementation regions, this contrast was not associated with increased activity in cerebellar-parietal regions as described in an earlier study using suggestion and PET to model ‘deluded passive’ movement [11]. If cerebellar-parietal activations are associated with the heightened awareness of unpredicted movements rather than the sense of their involuntariness, the absence of significant differences in the self-reported awareness of actions between the voluntary (hyp-vol) and involuntary (hyp-involA) conditions may account for the lack of modulation of cerebellar-parietal activity. This in turn may be related to the inclusion of a suggestion that “you will remain calm and relaxed during these movements of your hand” in each of the motor conditions. It is also notable that suggestions of reduced awareness of involuntary movements (hyp-involNA) relative to involuntary movements with preserved awareness (hyp-involA) was associated not only with reduced self-ratings of awareness but also with reduced parietal cortical activity. This provides additional support for the idea that modulation of parietal activity relates to the awareness of limb movements rather than the sense of their voluntariness. It may therefore be that the salience of unexpected limb movements associated with involuntary action may, where present, contribute to but not be necessary for the overall sense of the involuntariness of a movement. 

### Modelling neuropsychiatric and culturally influenced alterations in experience

This study demonstrates the potential of employing suggestion in association with fMRI to create experimental models or analogues of psychopathological and culturally influenced alterations in the control, ownership, and awareness of actions in the laboratory [39]. Each experimental hypnosis condition was preceded by a suggestion targeted to produce a specific alteration in the experience of movement which was then reversed at the end of each 5 minute scan. Changes in brain activation and / or connectivity associated with each condition are therefore likely to be involved in the accompanying alterations in the experience of movement and by analogy to the modelled clinical and cultural phenomena. However, it has also been postulated that pathological and culturally influenced dissociative symptoms in general, and dissociative seizures in particular, can be caused by suggestive and auto-suggestive processes [3,5,6,39,40]. The present experiment provides not only a working model of the putative brain changes that underpin relevant changes in experience but also the psychological process (suggestion) that helped produce them. Despite differences in form (e.g. external verbal commands in hypnosis versus non-verbal external or internal cues in dissociation), suggestive processes and their attributions appear to modulate relevant brain systems (e.g. motor regions in involuntary movements) via changes in otherwise intact prefrontal-executive control systems (e.g. the SMA complex) [41]. Similarly, passivity phenomena in schizophrenia (e.g. delusions of control) may be underpinned by functional changes in the SMA complex as modelled in the present experiment. However, this disconnectivity may result from dysregulation of executive and other brain systems associated with abnormalities of brain anatomy and neuromodulatory systems [42]. In this case, misattributions about the causes of actions may be secondary to disturbances of the sense of control, ownership, and awareness, rather than precede and contribute to them as occurs in the case of suggestions. The phenomenon of anarchic hand results from lesions that may also include medial prefrontal regions encompassing the SMA complex [1]. The present findings illustrate how altered function of the SMA complex [19] may result in a perceived loss of control and ownership of hand movements, indicating how loss or alteration of SMA function due to a lesion may disrupt the normal sense of control and ownership of the contralateral limb [1]. In summary, the current experimental model is relevant to understanding different forms of psychopathological and culturally influenced alterations of control, ownership, and awareness of limb movement when viewed in relation to the distinctive features of each phenomenon. 

The present experiment employed targeted suggestions and fMRI to investigate reduction of awareness of limb movement as reported in some psychopathological and culturally sanctioned dissociative phenomena. Reduced awareness of involuntary movements was associated with reductions in parietal cortex (BA 7, BA 40) and insula amongst other brain regions. This raises the question of whether comparable changes in brain activity accompany loss of awareness in anosognosia, a common and debilitating condition where neurological deficits are associated with differential and variable degrees of deficit unawareness including denial of disability in the case of hemiplegia, hemiparesis, and other disorders [43]. Targeted suggestions and fMRI could be employed to test this hypothesis. 

The study has a number of limitations. First, while we conducted our experimental protocol on 33 highly hypnotically suggestible volunteers outside the scanner, only 15 were suitable for and / or consented to participate in the fMRI experiment. Of these 15 individuals, 11 were included in the final analysis of motor activations (see Methods and Results). Further, only 7 were included in the connectivity analyses for the SMA seed. While these exclusions may potentially increase the risk of Type II errors by reducing the power to detect differences, we believe that it is more important to minimise the risk of Type 1 errors by robust checks on data quality. We would also add that because only approximately 10% of the population is highly hypnotically suggestible, we had to screen over 300 individuals to recruit the subset of individuals who eventually participated in fMRI scanning [30] – illustrating one of the practical challenges in modelling neuropsychiatric symptoms in highly hypnotically suggestible subjects. Future studies with larger sample sizes in our laboratory will be facilitated by the recruitment database (see Methods) and hypotheses established from the current study. 

We also found that the contrast of voluntary movement in the non-hypnosis condition (nhyp-vol) compared to voluntary movement following induction of hypnosis (hyp-vol) did not reveal significant differences in brain activity. This differs from the growing evidence that hypnosis is associated with modulation of default mode network (DMN) and attentional network activity [44]. Lack of modulation of DMN by the induction of hypnosis in the present experiment may be because simple guided motor output tends to produce limited task related deactivations, reducing the scope for modulatory effects of hypnosis on DMN activity to be detectable between conditions. However, we employed the voluntary movement following hypnosis condition (hyp-vol) in relevant contrasts to determine the effects of suggested involuntariness, so controlling for any potential effects of hypnosis *per se* on subtle differences in brain activity or connectivity. 

Experimental conditions were presented in a randomized order across participants, with the exception of a) the nhyp-vol condition, which always occurred prior to induction of hypnosis; and b) the hyp-vol condition, which was the first condition presented following hypnotic induction. The order of involuntary movement with (hyp-volA) and without awareness (hyp-volNA) was randomized and therefore differences between these conditions are unlikely to represent order effects. Following hypnosis the voluntary movement condition preceded the involuntary movement condition, although the timing of the latter condition was randomized. We note that a contrast of all of the other randomized conditions against hyp-vol did not show the same pattern of differences as hyp-vol vs hyp-involA – therefore, it seems unlikely that the specific results we observed for voluntariness can be explained as the result of a simple order effect. 

Prior studies have indicated a role for parietal regions in the sense of voluntary action [45]. However, we chose to focus on the SMA to avoid problems of multiple comparisons in view of our relatively small sample size and the well-established body of evidence supporting a role for SMA in the experience of voluntary movement [46]. Nevertheless, future studies with larger samples should investigate the relative contributions of the SMA complex and parietal cortices to the experience of voluntary and involuntary movement. Similarly, a future study with a larger sample should also investigate the relative contributions of feedforward and feedback signals to the experience and neural underpinnings of voluntary and involuntary movement – for example, by the inclusion of a passive movement condition as a contrast for suggested involuntary movement [11]. 

## Conclusion

The present study used targeted suggestions to investigate the functional anatomy of the voluntariness and awareness of movement, in healthy highly hypnotically suggestible participants. Compared to voluntary movements, involuntary movements were associated with reduced connectivity of SMA with other motor implementation regions. Modulation of SMA connectivity may therefore underpin loss of control and ownership of movements in pathological and culturally influenced dissociative phenomena, as well as delusions of alien control. Reduced awareness of involuntary movements was associated with reduced activity in parietal (BA 7, BA 40) and insula cortices, amongst other brain regions, suggesting a potential neural mechanism for the narrowing of awareness reported in pathological and culturally influenced dissociative phenomena. Thus, association cortices with somatotopic mapping (parietal and insular cortices) mediate awareness of limb movements, whereas the sense of voluntariness appears instead to depend on connectivity within motor system areas linking intention to action. Future studies could employ methods (such as EEG or MEG) to test the hypothesis arising from this study that the perceived control and ownership of actions is mediated by the correlated activity of SMA with motor implementation systems. 
